# Implementing a Commercial AI Fracture Detection Tool in Health Care Using the Non-Adoption, Abandonment, Scale-Up, Spread, and Sustainability Framework: A Formative Evaluation Study

**DOI:** 10.2196/72648

**Published:** 2026-06-26

**Authors:** Gro-Hilde Severinsen, Line Silsand, Gunnar Ellingsen, Rune Pedersen

**Affiliations:** 1 Norwegian Center for E-health Research, Sykehusveien 21, Tromsø, 9019, Norway, 47 90199457; 2 Faculty of Health Sciences, UiT The Arctic University of Norway, Tromsø, Norway; 3 Faculty of Nursing and Health Sciences, Nord University, Bodø, Norway

**Keywords:** formative process evaluation, Non-Adoption, Abandonment, Scale-up, Spread, and Sustainability, NASSS, information infrastructure, commercial AI, health care innovation

## Abstract

**Background:**

Artificial intelligence (AI) has the potential to enhance resource efficiency, improve patient treatment, and increase safety in health care. Still, there is limited knowledge on how to implement and evaluate AI solutions in real-world clinical settings. To address this gap, we conducted a formative process evaluation of the first large-scale procurement and implementation of a commercial AI solution in Norwegian health care. F The Non-Adoption, Abandonment, Scale-up, Spread, and Sustainability (NASSS) framework, was used for the formative process evaluation throughout the 4-year project to guide data collection, analysis, and real-time feedback.

**Objective:**

This study aimed to evaluate the usefulness of the NASSS framework for formative process evaluation of AI implementation in health care.

**Methods:**

A formative process evaluation was conducted from 2020 to 2024, covering the procurement, preimplementation, and implementation phases. Data included 65 interviews, observations, and document analysis. Data were analyzed thematically using the 7 NASSS domains, supplemented with subtopics within each domain to capture emerging infrastructural complexities and temporal dynamics. Real-time findings were discussed with the implementation team, decision-makers, and clinicians.

**Results:**

Key factors for successful implementation included clinician trust, workflow integration, task distribution, and digital maturity. Major challenges comprised limited documentation of Conformité Européenne–marked solutions, deskilling, and misaligned financial incentives. The NASSS framework enabled the identification of sociotechnical values and complexities, but did not fully capture workflow evolution and changing user perceptions over time.

**Conclusions:**

The NASSS framework is useful for evaluating AI implementation but requires adaptation to capture temporal dynamics and workflow changes better. These findings contribute to improving evaluation approaches for AI in health care.

## Introduction

This study reports on the first large-scale implementation of a commercial artificial intelligence (AI) solution in a large Norwegian health care trust in South Norway between 2020 and 2024. Vestre Viken Health Trust (VVHF) serves approximately 500,000 people across 22 municipalities and includes approximately 10,000 staff at 4 hospitals and 1 infirmary [1]. The imaging diagnostics department spans all 5 units. This paper describes the 5-year process evaluation of the procurement and implementation of the AI solution BoneView for fracture detection [2], using the Non-Adoption, Abandonment, Scale-up, Spread, and Sustainability (NASSS) framework [3,4]. In this paper, we strictly limit the term “AI” to commercially available Conformité Européenne (CE)–marked AI solutions [5,6].

Implementing AI solutions such as BoneView could potentially improve resource efficiency, patient treatment, and safety in health care [7-10]. The improvements include reducing diagnostic errors, optimizing clinical workflows, and decreasing waiting times. However, despite rapid technological development, most AI research focuses on model performance rather than implementation processes. Limited understanding of implementation leaves substantial knowledge gaps for health care organizations regarding the adoption of AI in real-world clinical settings [9,11-13]. Strengthening the evidence base for implementation is therefore essential to ensure the successful implementation of AI and the creation of long-term value for implementation projects, decision-makers, and health care professionals [9,14].

In 2020, a national inquiry led by the Directorate of Health in Norway identified opportunities and barriers to deploying AI in health care, emphasizing the need for real-world implementation to fully understand its impact [15]. The results aligned well with national strategies for digital transformation and responsible AI use in public services [10,16]. Meanwhile, VVHF, one of Norway’s largest health trusts, wanted to take a leading role in implementing AI in health care. Hence, a national pilot was commissioned for VVHF to procure and implement the first large-scale AI solution in Norwegian health care. This resulted in the procurement of an AI marketplace platform, including several AI solutions, starting with BoneView from the vendor Gleamer [2], a CE-marked AI solution for fracture detection in radiology.

The Directorate of Health also commissioned our research team at the Norwegian Centre for eHealth Research to conduct a formative process evaluation of the pilot project to start building a national knowledge base on the implementation of commercial AI solutions. Knowledge on large-scale AI implementations in health care was scarce at the time, both in Norway and across Europe, underscoring the need for an extensive prospective evaluation. Previous studies have identified significant sociotechnical challenges related to the adoption of AI, such as workflow disruption, low digital maturity in organizations, and a lack of trust in AI solutions. These studies have also highlighted an underdeveloped use of evaluation frameworks within AI research [11,16-18].

To address these issues, this study presents a formative process evaluation of the BoneView implementation at VVHF using the NASSS framework. Several studies of health care innovations use this framework, including a few AI applications [3,19,20]. However, reviews show that NASSS remains underused in AI implementation research [3,19,20].

Hence, this study has two aims: (1) to evaluate the usefulness of the NASSS framework for the process evaluation of AI implementation, and (2) to identify key sociotechnical barriers and facilitators across AI implementations in health care settings.

## Methods

### Study Design

A formative evaluation is a rigorous process designed to identify potential and actual influences on implementation efforts [21,22]. Formative process evaluation is the recommended approach for complex digital health interventions. It enables a systematic evaluation of the sociotechnical interplay among users, technology, and organizations across various implementation phases [10,21,23-25]. It therefore provides actionable insights into the process of deploying commercial AI in clinical practice [26]. This approach requires close collaboration between researchers and the implementation team. It is a research-driven, evidence-oriented approach in which data are collected and analyzed across the 3 project phases [21,23,25]. Findings are shared with the implementation project to support continuous improvement and surface emerging complexities. It focuses on evaluating the implementation process, generating evidence, and providing feedback, while researchers refrain from making decisions or shaping the intervention directly [21,23,25].

Implementing digital solutions, such as AI, is inherently messy and complex. Traditional research designs alone rarely capture the nuances required for an understanding of real-world implementation [21]. Rather than replacing conventional methods focused on effectiveness and cost-benefit outcomes, formative process evaluation complements them, creating a richer, more context-sensitive evidence base. In this project, this evaluation was essential for identifying specific barriers, values, and sociotechnical preconditions, and for supporting organizational learning.

The iCHECK-DH (Guidelines and Checklist for Reporting Results of Internet E-Surveys for Digital Health) guidelines structure the presentation of this study (refer to Checklist 1) [27]. The aim of using the checklist is to standardize the documentation of digital health implementations by ensuring transparency regarding context, implementation strategies, and influencing factors [28].

### The AI Solution and the Implementation

An important motivation for implementing AI solutions was the continuous 5%‐10% annual increase in imaging volume, without a proportional increase in staff [1]. Between 2020 and 2022, VVHF conducted a dialogue-based procurement process. The aim was to generate valuable insights on how to optimize AI implementations and understand their expected effects and values [26]. BoneView from Gleamer, for detecting x-ray fractures in radiology was selected as the first solution to be deployed [2,29]. BoneView was part of a Philips and Blackford AI marketplace platform. During the preimplementation phase (2022‐2023), VVHF conducted an extensive internal validation of 1600 images at the first hospital followed by about 300 images at the other hospitals. The output confidence levels are categorized as positive (>90%), doubtful (50%‐90%), or negative (<50%). BoneView was successfully deployed across all 5 VVHF units between August and December 2023 [26].

BoneView is a CE-marked AI solution for x-ray examinations used to identify fractures, bone lesions, hydrops, and dislocations in patients aged 2 years or older. The algorithm is strictly approved as a clinical decision support system and cannot make autonomous decisions [26,30]. The image is taken by radiographers and sent from the Picture Archiving and Communication System (PACS), anonymized, analyzed in a cloud-based AI solution based in Belgium, and then reidentified upon return to PACS, and visualized in the Radiology Information System (RIS) using a traffic light model for triage [30].

### Participants and Recruitment

Participant recruitment proceeded continuously throughout the 3 project phases. Initial participants were identified by the VVHF project group based on their involvement in the procurement and implementation work. These participants then facilitated the recruitment of leaders, radiologists, radiographers, and representatives from regional health authorities. Recruitment was unproblematic, and most contacted stakeholders agreed to participate. As a result, the final sample included a broad set of stakeholders across all VVHF hospitals and at the system level, enabling a multiperspective understanding of the project.

### Data Collection

**Figure 1.**
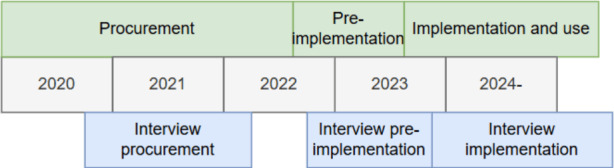
This is an overview of the 3 phases of the procurement and implementation project in Vestre Viken Health Trust, alongside the 3 interview phases in the process.

Data were collected from 2021 to 2024, including 3 phases of the procurement and implementation process (Figure 1).

The data comprised 1 focus group and 65 individual interviews, all conducted digitally via Microsoft Teams. Some stakeholders participated in 2 to 3 interviews throughout the process, which was valuable for tracking changes in attitudes and trust over time (Table 1).

**Table 1.** This is an overview of the data collection in the 3 phases of procuring and implementing the AI^a^ solution in VVHF^b^ (the table includes the number of stakeholders involved, the number of observation hours, a description of the meetings and workshops observed, and the number of hours spent on document studies).

Method	Procurement phase	Preimplementation	Implementation
Interview	19 informants 3 radiologists 11 leaders 5 other informants	9 informants 4 radiologists 2 radiology leaders 1 radiographer 1 leader 1 legal consultant	35 informants 11 radiographers 9 radiographers and radiology leaders 10 radiologists and orthopedics 5 other leaders
Observations	50-hour dialogue meetings with vendors	20 hours of observing workshops preparing hospitals for implementations	5 hours of observing radiographers using BoneView Observers in the regional network for AI radiology every third month
Meetings and workshops	Meetings with the project group every 3 weeks Evaluation in VV^c^ after the procurement	Meetings with the project group every 3 weeks Workshop with Blackford analysis Meetings with the Norwegian Directorate of Health every 3 weeks	Meetings with the project group and the Directorate of Health every 3 weeks
Document studies	10 hours	20 hours	50 hours

a AI: artificial intelligence.

b VVHF: Vestre Viken Health Trust.

c VV: Vestre Viken.

Our interview guide was a modified version of the NASSS interview guide, with necessary adjustments to align with Norwegian health care settings, and the diverse stakeholder groups. Interviews lasted between 30 and 90 minutes and began with participants sharing their expectations or experiences of the implementation process. All interviews were recorded with oral consent and conducted by 1 to 3 researchers. The research group transcribed, anonymized, and analyzed the data.

**Figure 2.**
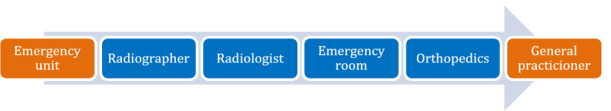
An overoview of the wide range of stakeholders involved in the fracture detection workflow.

The data collection also involved observing radiographers using the AI solution at 2 locations. We obtained written consent from each patient examined during these observations. We also observed several meetings and workshops, including vendor dialogue meetings during the procurement phase and postimplementation evaluation meetings, during which process improvements were discussed. Before implementing the AI solution, VVHF organized workshops at each hospital, with representatives from all stakeholders involved in the bone fracture pathway (Figure 2). They discussed when, why, and for whom the solution required workflow changes. Our research group met every 3 weeks with (1) the Directorate of Health, with whom we discussed national challenges relating to knowledge exchange and the governance of commercial AI solutions. (2) The project group in VVHF is implementing the AI solution. The latter group served as the key forum for discussing process evaluation results, ongoing activities, and maintaining an updated, shared understanding of the implementation process. We also participated as observers in the regional network for AI in radiology in the Southeast Health Region every 3 months.

### Analytical and Theoretical Framework

#### Use of the NASSS Framework

**Figure 3.**
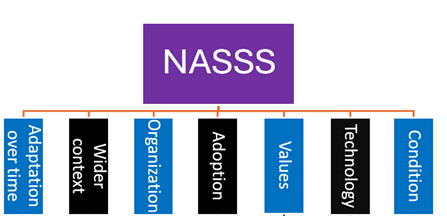
This is an overview of the 7 categories of the Non-Adoption, Abandonment, Scale-up, Spread, and Sustainability framework, including condition, technology, values, adoption, organization, wider context, and adaptation over time.

For the formative process evaluation, we chose the NASSS evaluation framework (Figure 3). NASSS was originally developed as a synthesis of 28 technology implementation models and was designed to reveal the structural and sociotechnical complexities that influence digital health projects, including adoption, nonadoption, abandonment, scaling, and sustainability [4,31]. NASSS has been successfully used in numerous digital health evaluations [12]. However, research shows that AI implementation studies often lack theoretical grounding and rarely apply NASSS prospectively, despite its potential to guide implementation processes. Our research group has previously used NASSS successfully to evaluate implementations of digital health care solutions [3,19,20]. In the VVHF study, NASSS was used prospectively throughout the 4-year evaluation process, guiding both data collection and analysis. It captures sociotechnical complexities across 7 domains: condition, technology, value, adoption, organization, wider context, and adaptation over time (Figure 3). NASSS is also increasingly used to study digital health implementations involving multiple stakeholders and infrastructure dependencies.

#### Information Infrastructure as a Theoretical Rationale

While NASSS structured the overall evaluation of this study, Information Infrastructure (II) theory was added to provide a valuable additional perspective on the stepwise, 4-year-long implementation process. II theory emphasizes that digital systems in health care are always embedded within an installed base of existing technologies, routines, professional roles, and institutional constraints [32-34]. This perspective closely aligned with our findings across the procurement, preimplementation, and implementation phases, in which BoneView had to integrate into a complex landscape comprising RIS, PACS, and electronic health record systems, established workflows, and regulatory requirements. II theory views digital health systems as evolving sociotechnical infrastructures that grow organically over time [32-34]. This emphasis on temporality and interdependence helped us to interpret dynamics that NASSS alone only partially captured. This includes how micro-level and macro-level governance structures shaped workflow practices. It also includes recognizing that the implementation required continuous adjustments as more stakeholders became involved. The inherent dynamism of infrastructure also explains why AI implementation generated ripple effects across the organization, influencing everything from clinical workflows and role expectations to trust, work trajectories, and digital maturity.

Key II concepts, such as installed base, bootstrapping (design for instant usefulness), and adaptation over time [32], clarified challenges that NASSS could only partially capture. These included the need to fit BoneView into an already complex infrastructure. Also, the dependency between new tools and existing workflows, and the importance of iterative adjustments, as more stakeholders became involved. The II theory also helped explain why even a simple, CE-marked “off the shelf” AI tool required significant sociotechnical work to become usable for clinical practice [32-34]. In this study, II theory is an interpretive supplement to NASSS, not a full parallel framework. By adding a temporal and infrastructural perspective, the II theory enriched the NASSS-guided evaluation. It provided deeper insight into how AI solutions become embedded in health care organizations and why successful implementation depends on ongoing negotiation, adaptation, and alignment across the system.

### Analysis

**Figure 4.**
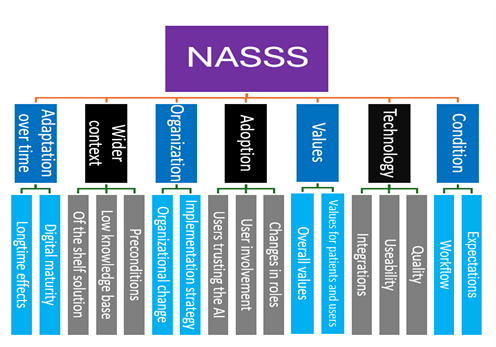
An overview of the 7 Non-Adoption, Abandonment, Scale-up, Spread, and Sustainability (NASSS) categories and the subtopics identified in the study to nuance the findings beyond what the Non-Adoption, Abandonment, Scale-up, Spread, and Sustainability frameworks could achieve.

Our data analysis included systematically reading the interview transcripts and deductively coding them into the 7 NASSS categories [3]. We used a hermeneutic approach to analyze all the data. This approach involves analyzing data from each project phase separately and the entire dataset together to achieve a balanced, comprehensive understanding of the findings [35]. However, the volume of data in each category was too large to identify key values and complexities at first glance. Therefore, we conducted a thematic analysis with inductive coding to identify 2‐3 subtopics within each domain (Figure 4) [36]. Discussions within the research team and with the project group in VVHF determined the final selection of subcategories within each NASSS category.

### Ethical Considerations

The Data Protection Officer (DPO) at University Hospital of North Norway determined that the project did not require approval from the Regional Committee for Medical and Health Research Ethics, as the processing of personal data did not fall under medical and health research under Section 2 of the Norwegian Health Research Act. The processing of personal data complied with the General Data Protection Regulation Articles 6(1)(a), 9(2)(a), 9(2)(j), and 89(1), and was documented in accordance with Article 30 of the General Data Protection Regulation. The project was registered under the University Hospital of North Norway DPO project number 02606VVHF and approved by the DPO at Vestre Viken (20/10471-1).

## Results

### Overview

The procurement of the AI platform, including the BoneView solution, was conducted by VVHF; however, all the other health trusts in the region (15 in total) had an option to purchase it, making it a regional project. BoneView progressed from the procurement phase (2020‐2022) and preimplementation phase (2022‐2023) to full deployment (late 2023) and initial scaling and adaptation (2024). The findings were categorized using the NASSS framework (Figure 4). The results from each category are presented below, outlining topics to be aware of regarding value creation and complexities during AI implementations. The evaluation identified crucial factors, including prerequisites for clinician trust and adoption of the solution, the impact on user workflows, digital maturity in implementing new technology, and considerations around legislation, validation, management, and workflow optimization. For an overview of the NASSS categories and the key findings, refer to Table 2 below.

**Table 2.** An overview of the 7 NASSS^a^ categories and the key findings and implications of each category^b^.

NASSS domain	Key findings	Implications
Condition	Fracture detection is low-risk and suitable for early AI^c^ use. Workflow efficiency improved with radiographer-led triage. Role expectations shifted differently for radiologists and radiographers.	Even simple clinical conditions require careful workflow redesign. Role changes must be supported with training and clear responsibilities.
Technology	High diagnostic performance but limitations in children, older adults, and distinguishing old from new fractures. Processing delay (1‐5 minutes) affected efficiency. Integrations with RIS^d^, PACS^e^, and EHR^f^ were incomplete.	Human oversight remains essential. Seamless technical integration is critical for adoption. Speed and reliability determine perceived usefulness.
Values	Major value for patients: reduced waiting time, fewer ER^g^ visits, immediate triage. Limited short-term workload reduction for radiologists. Benefits spread across departments, not only imaging.	Economic models must account for cross-department benefits. Patient value is a strong driver for AI implementation.
Adoption	Trust depended on extensive local validation (1600 images). Radiologists involved early; radiographers joined later despite major workflow changes. New responsibilities increased empowerment but also stress.	Early and continuous user involvement is key. Different professions require tailored onboarding. Training and support must match new responsibilities.
Organization	Bottom-up “learning by doing” strategy enabled iterative improvement. Broad stakeholder involvement fostered ownership. Organizational change was more challenging than technical integration.	Implementation strategies should emphasize cocreation and flexibility. Preimplementation workshops reduce resistance. Organizational readiness is as important as technological readiness.
Wider context	Limited national experience with AI procurement and implementation. GDPR^h^ compliance and European cloud hosting were essential. Dialogue-based procurement enabled better evaluation of vendor capabilities.	National-level guidance and standards are needed. Legal and regulatory complexity must be addressed early. “Off the shelf” AI still requires extensive local validation.
Adaptation over time	Improved digital maturity and readiness for future AI tools. Potential long-term redistribution of tasks to radiographers and orthopedics. Concerns about the impact on radiologist and radiographer training.	Long-term planning should include workforce development and training. Early-stage AI implementations can prepare organizations for more complex tools. Continuous evaluation is needed to balance efficiency and clinical competence.

a NASSS: Non-Adoption, Abandonment, Scale-up, Spread, and Sustainability.

b This overview provides an overview of the process evaluation conducted in Vestre Viken Health Trust. More details are presented in the subsections below, which describe the most important findings in the different Non-Adoption, Abandonment, Scale-up, Spread, and Sustainability categories.

c AI: artificial intelligence.

d RIS: Radiology Information System.

e PACS: Picture Archiving and Communication System.

f EHR: electronic health record.

g ER: emergency room.

h GDPR: General Data Protection Regulation.

### Category 1: Condition

The first NASSS category considers clinical condition, potential comorbidities, and sociocultural factors to determine whether patients are suitable candidates for this technology [3,4]. Rather than referring to a single condition, fracture detection encompasses the assessment of all types of fractures in the body. The only inclusion criterion is a suspected fracture requiring x-ray examination. As we did not have a specific condition to address, we used this category to highlight significant findings that did not fit into any other NASSS category. This included user expectations and attitudes, as well as the impact of AI solution implementation on image diagnostics workflows.

**Figure 5.**
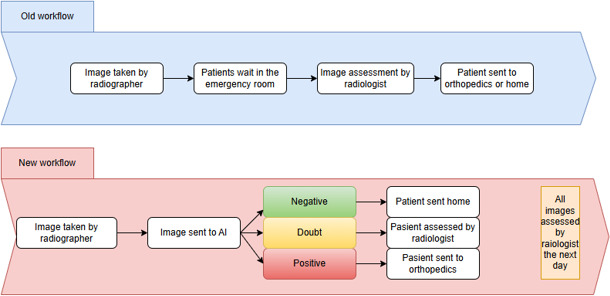
This is an illustration of the old workflow of imaging diagnostics befor using AI (blue) and the new workflowd of imaging diagnostics (pink) after AI was implemente. AI: artificial intelligence.

Overall, implementing BoneView has significantly optimized the fracture detection workflow. Although all the hospitals had different workflows before the AI solution, the overall workflow still included sending patients back to the emergency room to wait for radiologists to assess the images. This often resulted in the patients waiting for several hours. In the new workflow, radiographers using the AI solution assess images and send patients with positive results to orthopedics. Those with negative results are sent home, and those in which the AI solution is in doubt are sent for radiologist assessment, as before. Although using the AI solution has streamlined the fracture workflow, radiologists still assess all x-ray images the following day to ensure quality (refer to Figure 5 for the new and old workflows). This means that many patients are sent home without any waiting time.

The other subtopic in this category concerns attitudes and expectations regarding the use of an AI solution in image assessment. Radiologists initially approached the AI solution with cautious optimism. They gradually recognized its potential to enhance fracture detection and free up time for addressing more complex diagnostics. Some were skeptical about its immediate impact on clinical workflows and the diagnostic quality. Radiographers, on the other hand, were initially more optimistic and many embraced the expanded role in imaging assessment and patient distribution. Still, some expressed concerns about how using BoneView required them to spend more time on each patient and reshaped their responsibilities, without additional training or financial compensation.

The key takeaway from this category is that even simple clinical conditions still require substantial workflow and role adjustments when introducing AI.

### Category 2: Technology

This category addresses the material and technical features of the technology, usability, data, and knowledge generated or made visible by the technology, the knowledge and support needed to use the technology, and sustainability [3,4]. The 3 subtopics addressed in this category include quality, integration, and usability.

The quality of BoneView in relation to local images was a key issue to address for clinicians to trust it. A retrospective validation proved that BoneView was almost as effective as radiologists. Furthermore, BoneView detected some fractures that the radiologists missed, and performance was unaffected by fatigue and stress. Hence, radiologists reported on its potential to improve overall diagnostic quality. However, limitations such as the inability to account for medical history, distinguish between old and new fractures, and accurately interpret certain patient groups (eg, children and older adults) highlight the importance of human oversight in the diagnostic process. This means that the AI solution requires optimization, to be approved as for instance an autonomous and learning solution. There are both legal and trust barriers to using it autonomously. Radiologists say that the AI solution made too many mistakes to operate autonomously.

Integration and workflow efficiency are also important considerations when using the AI solution. Significant challenges were encountered, including delays in image analysis ranging from 1.10 to 5.00 minutes. Handling up to 60 x-rays in one shift, a 2‐ to 5-minute delay per patient adds up significantly across the day. Seamless integration with existing IT systems (eg, RIS, PACS, and electronic health record) is also critical to minimize disruption and ensure that the technology adds value without complicating the workflow or increasing the number of clicks or logons, especially if more than one AI solution is required.

The key takeaway from this category is that the effective use of AI requires high technological reliability, seamless integration, and well-designed user interaction.

### Category 3: Values

The third NASSS category considers the value of innovation and who benefits from it. This includes asking whether new technology is worth developing. It considers both the supply side and demand side values of health technology appraisal, as well as value for patients [3,4]. The subtopics highlighted in this category were user and patient values, as well as the organization’s overall financial value.

Regarding value for users, the main objective of the implementation was to free up time for radiologists, given the pressing shortage of clinical resources. Still, radiologists did not expect much value from the initial AI solution, since fractures were not the most pressing area, and they still had to assess all images. Nevertheless, the introduction of BoneView shifted roles between technology and users, generating immediate usefulness. Having the AI solution as a decision support tool was particularly beneficial for inexperienced radiologists and radiographers. One concern was the risk of overreliance on BoneView results and less trust in one’s own assessment. With the support of the AI solution, radiographers sent most patients with negative results home. This also led to fewer patients being sent to the emergency room, saving 15 consultations a day during the first 6 months of use. This change in workflow provided significant value to patients, as 50%‐60% of those referred to imaging diagnostics receive negative results. During the first 6 months of using BoneView, patients saved approximately 55 days of waiting time, and 1500 patients were sent home immediately.

To justify the long-term adoption of BoneView, it is necessary to consider the financial implications of its implementation from an overall perspective. The image diagnostics department covered the cost of investing in and deploying the AI solution; their direct benefits were limited in terms of time and cost savings. The emergency department and the patients gained the most benefits. To justify financial investment, we emphasize the need to consider the organization-wide benefits of using AI solutions. This involves discussing financial models and recognizing the ripple effects of AI implementations across the organization, which are essential for long-term sustainability. Furthermore, as AI implementations are monitored over time as an ongoing postimplementation process, long-term benefits for the imaging diagnostics department are expected to emerge.

The key takeaway from this category is the need to consider financial values at an organizational level, as well as the value of work distribution for radiologists and radiographers. Not having to wait for radiologists saved patients hours of waiting.

### Category 4: Adoption

The fourth NASSS category concerns the adoption and use of technology by patients and staff [3,4]. Our study focuses on health care workers’ adoption or abandonment of the BoneView solution. This category emphasizes building clinicians’ trust, changes in roles and relationships, and user involvement.

The most important success factor in implementing BoneView is building user trust in the solution’s quality. During the preimplementation phase, it became clear that users did not trust the AI solution. The most significant barriers were concerns about the solution’s overall quality and the lack of local validation [26]. Consequently, VVHF conducted extensive retrospective validation involving 1600 images at the first hospital. The results confirmed that BoneView achieved approximately the same sensitivity and specificity as reported by vendor documentation for traditional x-ray examinations. This rigorous process reassured users and demonstrated that the AI solution met local requirements and existing diagnostic standards.

Also, user involvement and continuous dialogue are essential preconditions for acceptance. Radiologists were therefore included at every step of the implementation process: this included identifying areas where AI solutions were useful for decision support. They participated in vendor dialogue meetings during the procurement phase and preimplementation workshops mapping workflows. They validated and tested the solution. Nevertheless, the process was lengthy, and only a few radiologists could participate. This led to varied perceptions of the process: some radiologists felt prepared and informed, while others felt less included. The radiographers were not consulted before the solution was implemented. This was challenging, since the radiographers were the main users of the AI solution.

The final subtopic in this category was changes in roles and responsibilities. Using BoneView enabled temporarily transferring tasks from radiologists to radiographers. Radiographers now play a more active role in assessing images, patient distribution, and providing follow-up advice. They send most patients with negative AI results home without waiting. They highlighted the need for updated training and compensation for enhanced responsibility. Radiologists supported the redistribution of tasks, since they still assessed all images the following day. Implementing the AI solution improved radiologists’ workflow efficiency, enabling them to focus on complex cases and leave fracture assessments to the next day.

The key takeaway from this category is the importance of gaining users’ trust in the AI solution through extensive validation and user involvement.

### Category 5: The Organization

The fifth NASSS category concerns preparing organizations for innovation. This includes the capacity to embrace service-level innovations, the readiness for specific technologies, and the interdependencies between organizations [3,4]. In this category, we discuss VVHF’s implementation strategy, emphasizing the importance of organizational change to succeed with AI implementations.

The most common approach to large-scale implementation in health care is a top-down strategy, where technology is implemented simultaneously across an organization; this is a complex, time-consuming process. However, VVHF opted for a bottom-up “learning by doing” implementation strategy, focusing on one hospital at a time. To address new preconditions for implementation, such as cloud storage, technical integration, legislation, and validation efficiently, relevant stakeholders, including technologists, lawyers, and clinicians, were included in the project group. This generated understanding, ownership, and a more efficient process. Rather than spending time writing strategies and plans, VVHF identified the key goals and requirements for implementing the AI solution, documenting and evaluating each step of the process successively. They then reused and improved the documentation for each implementation.

The project group collaborated closely with the change management team to inform staff and prepare them for the new technology. Including an organizational change process alongside the implementation ensured that the AI solution met organizational and clinical requirements. Implementing AI solutions in health care requires more than just deploying technology; organizational restructuring and workflow adaptation are also necessary, which can be challenging given the numerous stakeholders involved in fracture detection across different levels of the health care system (Figure 2).

Thanks to VVHF’s collaborative approach and the focus on change management, these challenges were addressed systematically. An important part of the process was holding preimplementation workshops with all relevant stakeholders at each hospital (Figure 2). The workshop prepared them for changes in workflows, reducing resistance, and confirmed alignment across different health care levels. A radiography leader said that improving efficiency depends on organizational factors and restructuring current workflows as much as on the technology, since BoneView is a static AI solution, and extensive alterations to the solution require new CE marking.

The key takeaway from this category is that organizational change was essential for aligning workflows, expectations, and responsibilities. The AI implementation strategy included a bottom-up approach and a cross-disciplinary project group.

### Category 6: Wider Context

This category addresses the simplicity or complexity of the wider institutional and sociocultural context of innovation [3,4]. The subtopics address the lack of a knowledge base and the implementation of commercial “off the shelf” AI solutions.

Due to a lack of knowledge and evidence-based guidance on procuring and implementing commercial AI solutions, VVHF decided to use a dialogue-based procurement approach. This was the first time this approach was used for health care innovations in Norway. They started with a short proposal inviting vendors to participate. The next step was extensive dialogue with each qualified vendor to establish a thorough understanding of their AI solutions. They then finalized the purchase document and selected the vendor. This process made it possible to better getting to know each vendor, still it was a complex and time consumin to conduct. During this process, VVHF also opted to shift from procuring individual algorithms to acquiring an AI platform. Choosing a platform provides flexibility to test and replace different solutions without initiating new complex procurement processes.

The final challenge in this category was challenges with using BoneView as an “off the shelf” ready for implementation. BoneView had the most robust clinical documentation and was already deployed in several European health care settings. Still, the documentation on clinical quality was insufficient, which generated mistrust among clinicians and hindered immediate deployment. Local validation became essential to build trust among clinicians.

The key takeaway from this category is that implementing commercial AI in health care is less about technical deployment and more about a complex organizational process. This process requires new procurement approaches, workflow adaptations, local validation, and trust building to make “off the shelf” solutions clinically usable.

### Category 7: Adaptation Over Time

The seventh category focuses on the adaptation and coevolution of technology and service delivery. In this category, we focus on the pathway to digital maturity and the long-term effects of deploying AI solutions [3,4].

The initial deployment of BoneView for fracture detection was a strategic decision to introduce AI solutions in a low-risk area to foster digital maturity within the organization. Even if BoneView sometimes would miss a fracture, the consequences for patient outcomes were low if patients were sent home one day and then recalled the next day after the radiologist reviewed the image. BoneView provided immediate organizational benefits, including reduced patient waiting times and enhanced digital maturity. The implementation created a solid foundation for implementing more advanced AI solutions in high-risk clinical areas in the future.

The evaluation of BoneView highlighted two potential long-term effects: (1) enabling orthopedic specialists to assess positive fracture images with AI assistance, and (2) enabling radiographers to discharge patients without requiring radiologist oversight for specific types of fractures, supported by BoneView. If these studies confirm BoneView’s effectiveness, significant radiology resources can be saved. Radiologists hope that future AI solutions will free them from labor-intensive and tedious routine examinations. Although AI solutions can reduce workload, radiologists expressed concerns about the potential skill atrophy of new radiologists. Reduced exposure to routine examinations could lead to deskilling, underscoring the importance of careful attention to the future quality of radiologist education. Radiographers’ education also needs to be evaluated to account for new tasks and responsibilities arising from the introduction of AI .

The key takeaway from this category is that AI could potentially save radiologists time in the long run. BoneView increased digital maturity and prepared for using more advanced AI solutions. It also increases requirements for alterations in both the radiographer and radiologist professions.

## Discussion

### Principal Findings

This formative process evaluation had two aims: (1) to examine the usefulness of the NASSS framework for evaluating the implementation of a commercial AI solution and (2) to identify key sociotechnical values, barriers, and facilitators shaping AI adoption in health care.

In relation to (1) the usefulness of NASSS for process evaluation of AI implementation, we found that applied prospectively, NASSS supported real-time learning by surfacing sociotechnical preconditions to succeed that were impossible to capture by technical performance metrics alone. The use of NASSS also supports real-time learning through continuous dialogue between the research group and the project team. The findings were directly used to inform decisions in different parts of the implementation process. One example was how our preimplementation interviews with radiologists outlined the need for local validation to build trust. This included both confirming the quality of the AI solution as well as defining how well it fitted the local context. However, our findings also show that NASSS has limitations in capturing temporal dynamics, workflow evolution, and emerging infrastructural dependencies central to AI implementations. Complementing NASSS with an information infrastructure perspective improved sensitivity to these aspects. Also, it was difficult to use NASSS for managing the large volumes of heterogeneous data in this 4-year implementation process. In practice, the data in each NASSS domain were divided into 2‐3 subtopics to enable outlining values and complexities. Hence, it is necessary to make some adjustments to make NASSS an optimal framework for evaluating AI implementations in health care.

In relation to (2) sociotechnical values, facilitators, and barriers, using NASSS to evaluate the implementation of BoneView generated values at the organizational, user, and system levels. Key benefits included improved workflows, reduced waiting times, fewer emergency department consultations, and redistribution of triage tasks to radiographers. Other values included trust building, systematic user involvement, extensive change management focus, and building digital maturity stepwise. Barriers included challenges related to role negotiation, professional training, and concerns about deskilling, infrastructural integration, and misalignment between organizational costs and benefits. In addition, large-scale implementation of AI solutions had never been done before; therefore, the process included an extensive focus on addressing new legal and privacy protection issues, internal change management, technical integration, ethical considerations, discussing financial models, and knowledge governance as well as defining how to monitor the solution over time.

### Comparison to Prior Work

This study contributes to and extends a growing body of literature emphasizing the need for sociotechnical evaluations when implementing digital health technologies in general and AI solutions especially. Previous studies have shown that many AI implementation failures arise from insufficient sociotechnical oversight rather than technical shortcomings [3,37,38]. Our findings support this and align with prior studies by, for example, Greenhalgh et al [3,4] and Cresswell et al [9,14], reinforcing that implementation challenges arise primarily from interactions between technology, professional roles, and organizational structures rather than from algorithmic performance alone.

As an extension of the need for sociotechnical focus when evaluating AI implementation, our study demonstrates that even a relatively simple, off-the-shelf AI solution such as BoneView generated extensive ripple effects. They include issues such as workflows, value creation, trust, change management, digital maturity, and monitoring practices. These impacts are often overlooked by traditional performance-focused or cost-benefit evaluations [9,17], supporting calls for formative and process-oriented evaluation approaches that capture the broader implementation context and infrastructural conditions that include the installed base as the starting point for such implementations [9,14,32].

In line with prior studies highlighting the lack of dedicated frameworks for AI implementation evaluations in health care [2], this study presents an example of using the NASSS framework to outline how useful this framework is for evaluating AI implementation. This is an extension of existing research where NASSS is used for AI evaluation since we used the framework prospectively across procurement, preimplementation, and implementation phases. NASSS has most commonly been used retrospectively and [14,31] only rarely for AI-focused evaluations [11,12,31]; our approach responds directly to calls for concurrent and forward-looking evaluations that allow emerging tensions, values, and unintended consequences to be identified in ongoing implementations [31]. Our findings also engage with ongoing methodological discussions concerning NASSS. Echoing critiques raised by Greenhalgh et al [3], we experienced challenges related to overlapping domains and the analytical “messiness.” However, consistent with prior arguments, our results suggest that this conceptual openness is a strength in formative evaluation, enabling complex, interdependent sociotechnical dynamics to be surfaced rather than prematurely simplified. At the same time, our experience extends previous work by illustrating how domain overlaps and category granulation complicate longitudinal analysis, particularly when technologies incrementally reshape workflows and professional practices over time [23,31]. A new finding in this paper related to prospective use of NASSS was the need for granulating the findings to outline complexities and values, at the same time as nuancing the data made it challenging to capture the temporal aspect of the process. Still, the prospective evaluation enabled continuous feedback to the implementation project, with findings that contributed to adjustments along the way. This is in line with the intention of NASSS [3,4] and formative process evaluation [9,21,22,39].

Rather than discussing all findings individually, trust serves as an illustrative example of how our findings both align with and extend prior research. Consistent with earlier studies, we found that trust in AI systems is socially and organizationally produced rather than a consequence solely of regulatory approval or vendor assurances [9,11,12,17,18]. However, our findings extend this work by showing how trust operated not only as a prerequisite for adoption but as a longitudinal mechanism shaping workflow redesign, role renegotiation, and organizational value creation across multiple NASSS domains. This perspective aligns with II theory, particularly the emphasis on building the installed base to enable adaptation and long-term sustainability [32]. In our study, incremental trust building around AI-supported fracture detection contributed to a broader shift in professional attitudes, moving radiologists from initial skepticism toward increased openness to more advanced AI applications. This supports recent calls to conceptualize trust and adoption as evolving sociotechnical processes rather than static determinants [9,14], while also highlighting limitations of applying relatively static frameworks such as NASSS to capture such temporal dynamics.

Taken together, our findings corroborate and extend prior research advocating for formative, sociotechnical-informed evaluations of AI in health care. By applying NASSS prospectively and empirically demonstrating how values, trust, and complexity emerge and interact over time, this study contributes nuanced insights into the conditions under which AI implementations may succeed or fail in real-world clinical settings.

### Limitations

This study focused on implementing a single, low-risk CE-marked AI solution for fracture detection across one health care organization and one clinical domain. Because diagnostic errors in this fracture detection have limited clinical consequences and radiologists continue to review all images, the transferability of findings to higher-risk, autonomous, or learning AI systems is limited.

Although the formative and longitudinal design enabled real-time insight during implementation, the study captures only early adaptation and initial embedding, rather than long-term effects, spread, or sustainability. Potential consequences related to professional role evolution, training needs, and deskilling among radiologists and radiographers therefore remain insufficiently explored.

### Conclusion

Overall, the study highlights the importance of choosing formative evaluation approaches that can capture both sociotechnical complexity and temporal change over time. Strengthening formative, framework-guided evaluations will be essential to support safe, scalable, and sustainable adoption of commercial AI in health care. When applied reflexively and enriched with greater temporal sensitivity, NASSS offers a robust basis for guiding, interpreting, and learning from long-term AI implementation processes.

Future studies should include comparative, prospective evaluations of multiple commercial AI solutions across clinical domains, organizational contexts, and risk profiles. They should also incorporate patient perspectives, governance, financing, and ethics to outline an even broader set of values and complexities. Methodological work should further refine AI evaluation frameworks such as NASSS by strengthening temporal sensitivity and explicitly incorporating AI-specific and infrastructural considerations. Other frameworks should be tried out for similar evaluations since using NASSS as the primary analytical framework, supplemented by II theory analysis, may have limited the analytical scope; other frameworks could highlight additional dimensions of AI implementation not addressed here.

## Supplementary material

10.2196/72648 Checklist 1 iCHECK-DH guidelines.

## Abbreviations

AI: artificial intelligence
CE: Conformité Européenne
DPO: Data Protection Officer
iCHECK-DH: Guidelines and Checklist for Reporting Results of Internet E-Surveys for Digital Health
II: Information Infrastructure
NASSS: Non-adoption, Abandonment, Scale-up, Spread, and Sustainability
PACS: Picture archiving and communication system
RIS: Radiology information system
VVHF: Vestre Viken Health Trust
